# Dietary supplementation with free methionine or methionine dipeptide mitigates intestinal oxidative stress induced by *Eimeria* spp. challenge in broiler chickens

**DOI:** 10.1186/s40104-019-0353-6

**Published:** 2019-06-27

**Authors:** Angélica de Souza Khatlab, Ana Paula Del Vesco, Adhemar Rodrigues de Oliveira Neto, Roberta Pereira Miranda Fernandes, Eliane Gasparino

**Affiliations:** 1 Animal Science Department, State University of Maringá, Colombo Avenue, 5790, Jardim Universitário, Maringá, Paraná 87020-900 Brazil; 2 Animal Science Department, Federal University of Sergipe, Marechal Rondon Avenue, S/N, Jardim Rosa Elze, São Cristóvão, Sergipe 49100-000 Brazil; 3 EVONIK of Brazil, Arquiteto Olavo Redig de Campos Street, 105, Tower A, São Paulo, SP 04711-904 Brazil; 4 Physiology Departament, Federal University of Sergipe, Marechal Rondon Avenue, S/N, Jardim Rosa Elze, São Cristóvão, Sergipe 49100-000 Brazil

**Keywords:** Antioxidant, Coccidiosis, Gut, *INFG*, *PEPT1*

## Abstract

**Background:**

This study evaluated the effects of *Eimeria* spp. challenge and dietary supplementation with free methionine or methionine dipeptide on animal performance; expression of genes associated with the immune system, antioxidant system, and amino acid transport in the jejunum; and redox status of the jejunum of broiler chickens.

**Methods:**

A randomized, 2 × 3 factorial design was used, in which *Eimeria* spp*.* challenge was the first factor (*Eimeria*-challenged, EC, or unchallenged, UC, broilers) and methionine supplementation was the second factor (non-supplemented, NS; free *dl*-methionine, *dl*-Met; and methionine dipeptide, *dl*-methionyl-*dl*-methionine, *dl*-MMet). At 14 days of age, chickens were inoculated orally with sporulated oocysts of *Eimeria acervulina*, *Eimeria praecox*, *Eimeria maxima*, and *Eimeria mitis*. Birds were killed by cervical dislocation 144 h post-inoculation (PI), and the jejunum was collected for biochemical and molecular analyses.

**Results:**

EC broilers had a 13% lower feed intake (FI), 37% lower body weight gain (BWG), and 39% higher feed conversion ratio (FCR) than UC broilers. Chickens fed the *dl*-Met diet had higher BWG (about 12% higher) and better FCR (about 12% lower) than chickens fed the NS diet. EC chickens had lower relative weight of the bursa of Fabricius (51.8%) and higher relative weights of the spleen and whole intestine (53.6% and 26.3%, respectively) than UC chickens. *Eimeria* spp. challenge led to an increase in the levels of oxidative substances, such as nitrite and thiobarbituric acid reactive substances (TBARS), in the jejunum of chickens 144 h PI. Among UC chickens, those fed the *dl*-Met diet had higher total antioxidant capacity (TAC) and lower catalase (CAT) and superoxide dismutase (SOD) activities. EC chickens that received the NS diet had higher carbonylated protein content (CP). This result was associated with their lower TAC and catalase activity. The lower TAC in EC chickens might have been due to reduced expression of catalase (*CAT*) and superoxide dismutase 1 (*SOD1*) genes. Chickens fed the *dl*-Met and *dl*-MMet diets had lower nitrite content. *Eimeria* spp. challenge suppressed neutral amino acid transporter 1 (*B*^*0*^*AT1)*, peptide transporter 1 (*PEPT1)*, toll-like receptor 5 (*TLR5)*, interleukin 2 (*IL2)*, and occludin (*OCLN)* gene expression and enhanced cationic amino acid transporter 1 (*CAT-1)* and interferon gamma (*IFNG)* gene expression. The highest *PEPT1* expression level was observed in broilers fed the *dl*-MMet diet, and the lowest *TLR5* expression level was found in broilers fed the NS diet.

**Conclusion:**

Our results show for the first time that supplementation with methionine as free amino acid or dipeptide helps protect the intestinal cells of broilers under *Eimeria* spp. challenge from the oxidative damage induced by free radicals, mainly through modulation of the antioxidant system.

## Background

Coccidiosis, an intestinal infection caused by several species of coccidian protozoa of the genus *Eimeria* [1], can disturb metabolic and physiological homeostasis in broilers. During the infection, the immune [1] and antioxidant systems are activated [2], and alterations in nutrient digestion and absorption occur [3–5]. Several studies reported that animals with coccidiosis show changes in intestinal morphology [6, 7], alterations in the expression of genes encoding digestive enzymes and transport proteins in the small intestine [5, 8], increased formation of reactive oxygen species (ROS) and reactive nitrogen species (RNS) [9], alterations in antioxidant enzyme activities [2, 10], and reduced concentrations of non-enzymatic antioxidants [11]. These changes are associated with poor animal performance, low efficiency, and increased mortality, causing global economic losses every year [12]. Although various anticoccidial drugs are available, they are not able to completely eliminate the disease. Alternative products have been tested for their immune stimulating, anti-inflammatory, and antioxidant properties [13]. Many antioxidants are used as supplements in poultry diets. Methionine, the first-limiting amino acid in corn and soybean meal broiler diets, has been highlighted as an important nutrient for the immune system [14] and antioxidant defense system [15]. Because of the crucial roles of methionine in physiological processes, *dl*-methionine, *l*-methionine, and methionine hydroxy analog (MHA) have been used for dietary supplementation. Methionine dipeptide has also been used as a dietary supplement [16, 17].

Because it is a small molecule, methionine dipeptide is absorbed mainly by peptide transporter 1 (PepT 1), present in the enterocyte membranes, whereas free amino acids are absorbed by specifics transporters [18]. Some studies have shown differences in the absorption efficiencies of dipeptides and free amino acids [17, 19]. Small peptides may be faster absorbed, as PepT 1 has a higher *V*_max_ than the transport systems used by free amino acids [20]. In addition, competition for the same transporter could reduce the absorption efficiency of specific amino acids [21]. Another advantage of dipeptides over free amino acids is that their absorption is less affected by intestinal mucosal lesions [22]. In previous studies, we showed the antioxidant effects of supplementation with MHA or *dl*-methionine in heat stress-exposed broilers [23, 24]. In this study, we hypothesize that methionine supplementation can help alleviate the negative effects of *Eimeria* spp. challenge, and we test whether there are differences between the effects of free methionine supplementation and methionine dipeptide supplementation. In the case of diseases associated with reduced intestinal absorption, the administration of di- or tripeptides could be a protective factor against protein malnutrition [21]. Despite the importance of small peptides to animal health, few studies have investigated dipeptide supplementation. To the best of our knowledge, this is the first study reporting the biochemical and molecular effects of methionine dipeptide supplementation in broilers challenged with *Eimeria* spp.

## Methods

This study was approved by the Ethics Committee on Animal Use (CEUA No. 4000170615) of the State University of Maringá, Brazil.

### Animals and experimental design

A total of 384 one-day-old unvaccinated Cobb 500 male broilers were used. The chicks were raised in a temperature-controlled environment at an initial temperature of 33 °C with 24 h of artificial light per day. The temperature was gradually reduced according to bird age, as recommended by Cobb 500 management guidelines. Birds were housed in raised floor cages of 1.0 m^2^ (8 chickens per cage). Chicks were raised conventionally up to 10 days of age, after which they were reared following a completely randomized, 2 × 3 factorial design with eight replicates of eight birds per treatment. The first factor was *Eimeria* spp. challenge (*Eimeria-*challenged, EC, or unchallenged, UC, broilers) and the second factor was methionine supplementation (non-supplemented, NS; free *dl*-methionine, *dl*-Met; and *dl*-methionyl-*dl*-methionine, dl-MMet). Before birds were submitted to the respective treatments, they were fasted for 6 h and weighed.

At 14 days of age, one group of chicks from each diet treatment (*n* = 64) were inoculated orally with 1 mL of a solution containing sporulated oocysts of *Eimeria* spp. (EC group; 2 × 10^4^ *Eimeria acervulina*, 2 × 10^4^ *Eimeria praecox*, 1.6 × 10^4^ *Eimeria maxima*, and 4 × 10^4^ *Eimeria mitis*). UC chickens received orally 1 mL of saline solution. Each group was housed separately to avoid cross-infection. All birds were killed by cervical dislocation 144 h post-inoculation (PI) at 20 days of age. Birds had free access to water and feed throughout the experimental period. Experimental diets containing corn and soybean meal were formulated according to Rostagno et al. [25], and no anticoccidial drugs were added to the diets. Diet composition is shown in Table 1, and analyzed and calculated nutrient compositions are shown in Table 2.

**Table 1:** Composition of experimental diets, %, as is, fed to 10–20 days old broilers

Ingredient, kg	NS^a^	*dl*-Met	*dl*-MMet
Corn (7.8%)	54.89	54.89	54.89
Soybean meal (46%)	37.30	37.30	37.30
Soybean oil	3.80	3.80	3.80
*L*-Lysine HCl (78%)	0.16	0.16	0.16
*L*-Threonine (98.5%)	0.04	0.04	0.04
*dl*-Methionyl-*dl*-Methionine (95%, dipeptide)	–	–	0.29
*dl*-Methionine (99%)	–	0.28	–
Dicalcium phosphate (20%)	1.53	1.53	1.53
Limestone (38%)	1.16	1.16	1.16
Salt	0.45	0.45	0.45
Vitamin–mineral premix^b^	0.40	0.40	0.40
Inert filler	0.30	0.02	0.01
Total	100.00	100.00	100.00

^a^*NS* Non-supplemented diet (control diet), *dl-Met* diet supplemented with *dl*-methionine 99%, *dl-MMet* diet supplemented with *dl*-methionyl-*dl*-methionine 95%

^b^The diets supplied the following compounds (per kg): retinyl acetate, 3.44 mg; cholecalciferol, 50 μg; *dl*-α-tocopherol, 15 mg; thiamine, 1.63 mg; riboflavin, 4.9 mg; pyridoxine, 3.26 mg; cyanocobalamin, 12 μg; *d*-pantothenic acid, 9.8 mg; *d*-biotin, 0.1 mg; menadione, 2.4 mg; folic acid, 0.82 mg; niacinamide, 35 mg; selenium, 0.2 mg; iron, 35 mg; copper, 8 mg; manganese, 60 mg; zinc, 50 mg; iodine, 1 mg; and butylated hydroxy toluene, 80 mg

**Table 2:** Calculated and analyzed nutrient composition, g/kg, as is, of experimental diets fed to 10–20 days old broilers

Nutrients	NS^a^	*dl*-Met	*dl*-MMet
Analyzed composition^b^, g/kg			
Crude protein	217	220	216
Lysine	12.87 (100)^c^	12.78 (100)	12.59 (100)
Methionine	3.05 (24)	5.78 (45)	5.84 (46)
Methionine + Cystine	6.53 (51)	9.10 (71)	9.17 (73)
Threonine	8.54 (66)	8.73 (68)	8.41 (67)
Tryptophan	2.81 (22)	2.85 (22)	2.79 (22)
Valine	10.39 (81)	10.56 (83)	10.12 (80)
Isoleucine	9.56 (74)	9.75 (76)	9.27 (74)
Arginine	14.52 (113)	14.89 (117)	14.34 (114)
Calculated composition			
AME^d^, kcal/kg	3053	3052	3052
Calcium, g/kg	8.76	8.76	8.76
Available phosphorus, g/kg	4.50	4.50	4.50
Sodium, g/kg	2.00	2.00	2.00

^a^*NS* Non-supplemented diet (control diet), *dl-Met* diet supplemented with *dl*-methionine 99%, *dl-MMet* diet supplemented with *dl*-methionyl-*dl*-methionine 95%

^b^Feeds were formulated using the total amino acid contents of corn and soybean meal determined by near infrared reflectance spectroscopy. The total amino acid and other nutrient values are shown as gram per kilogram and not as a percentage of the diets. Total amino acid compositions were analytically determined by Evonik Industries (Hanau, Germany) using wet chemistry (high-performance liquid chromatography)

^c^Values in parentheses indicate the amino acid-to-lysine ratios (ideal protein concept)

^d^*AME* Apparent metabolizable energy

### Coprological analysis for coccidiosis diagnosis and histological analysis of the duodenum and jejunum

A pool of fresh excreta samples was randomly withdrawn from the cages of EC animals, and another pool was withdrawn from the cages of UC animals, 144 h PI. Coprological analysis was performed for the qualitative detection (presence or absence) of oocysts in excreta, as described by Gordon and Whitlock [26] with modifications. Approximately 2 g of feces was dissolved in 15 mL of distilled water and centrifuged at 2500 r/min for 2 min. The supernatant was discarded, and the pellet was dissolved in 10 mL of sucrose solution (density 1.18). This mixture was centrifuged again at 2500 r/min for 2 min. Then, the material was placed on a histological slide for oocyst detection. An Olympus BX50 Optical P1 microscope coupled to an Olympus PMC 35 B camera (40× objective lens) was used for visual analysis.

Duodenum and jejunum samples were collected immediately after slaughter (144 h PI) for histological analysis. Samples were cut longitudinally, carefully washed with cold sterile physiological saline, and fixed in Bouin’s solution for 6 h. After fixation, samples were dehydrated through a graded ethanol series, diaphanized in xylol, and embedded in paraffin. Semi-serial histological sections of 3 μm thickness were obtained. Samples were stained with hematoxylin-eosin, and histological images were captured using the Olympus BX 50 P1 Optical microscope coupled to the Olympus PMC 35 B camera (4× and 40× objective lenses).

### Animal performance and relative weight of organs

Animals were weighed at days 14 and 20 after a 6-hour fasting period, during which they had free access to water. For performance evaluation, each cage (eight birds per cage, *n* = 8) was considered an experimental unit. Feed intake (FI) was calculated as the amount of feed offered at the beginning of the experimental period (day 14) minus the feed residue at the end of the experimental period (day 20). Body weight gain (BWG) was calculated by the difference between the mean initial weight (day 14) and mean final weight (day 20). Feed conversion ratio (FCR) was calculated as the ratio of FI to BWG.

For analysis of relative organ weight, each bird was considered an experimental unit (*n* = 8), birds were selected according on the body weight average of each replicate. At 20 days of age (144 h PI), chickens were fasted for 6 h and then killed by cervical dislocation. The liver, whole intestine, spleen, and bursa of Fabricius were collected and weighed. The relative weight of the organs was calculated using the equation organ weight/body weight × 100.

### Analysis of the redox state of the jejunum

Six birds from each treatment, chosen on the basis of the average body weight of each replicate group, were killed by cervical dislocation 144 h PI. The jejunum was collected, washed with cold sterile physiological saline, frozen in liquid nitrogen, and stored at − 80 °C until analysis.

### Sample preparation

Nitrite (NO_2_^−^) content, catalase (CAT) activity, superoxide dismutase (SOD) activity, and thiobarbituric acid reactive substances (TBARS) content were determined. Briefly, 100 mg of jejunum was added to 1000 μL of 0.1 mol/L potassium phosphate buffer, pH 7.4. The solution was homogenized using a Van Potter homogenizer until complete dissociation and centrifuged at 10,000×*g* for 10 min at 4 °C. The supernatant was collected in a clean eppendorf tube and used as a sample.

For analysis of carbonylated proteins (CP), 200 mg of jejunum was added to 1000 μL of 0.05 mol/L phosphate buffer with 0.001 mol/L ethylenediaminetetraacetic acid (EDTA), pH 6.7. The solution was homogenized using a Van Potter homogenizer until complete dissociation. Then, the homogenate was centrifuged at 10,000×*g* for 10 min at 4 °C. The supernatant was collected in a clean eppendorf tube and used as a sample.

### Analysis of CAT and SOD activities

CAT activity was assessed by evaluating the ability of the enzyme to convert hydrogen peroxide (H_2_O_2_) to water and molecular oxygen. Twenty microliters of supernatant was added to 980 μL of the reaction mixture (1 mol/L Tris buffer containing 0.005 mol/L EDTA, pH 8.0, and H_2_O_2_). Enzyme activity was monitored using an Evolution™ 300 UV-VIS spectrophotometer (Thermo Fisher Scientific™), and readings were performed at 240 nm for 60 s. CAT activity was expressed as the amount of H_2_O_2_ consumed per minute per milligram of protein (ε = 33.33 mol/L^.^cm) [27].

SOD activity was measured as the ability of the enzyme to inhibit the autoxidation of pyrogallol, which generates superoxide anions (O_2_^−^•). SOD competes with the detection system for O_2_^−^•. The absorbance increase was measured at 420 nm for 180 s using a microplate reader (VersaMax™, Molecular Devices). The jejunum supernatant was added to 0.2 mol/L Tris-HCl buffer containing 0.002 mol/L EDTA, pH 8.2, and 0.015 mol/L pyrogallol. The analysis was performed in duplicate at room temperature. A unit of SOD (U) was defined as the amount of enzyme required to inhibit the autoxidation rate of pyrogallol by 50%. SOD activity was expressed as U/mg of protein [28].

### Determination of biomarkers of oxidative stress and total antioxidant capacity (TAC)

Analysis of NO_2_^−^, one of the two stable and non-volatile primary products of nitric oxide degradation, was performed to assess the formation of nitric oxide during infection, as it is a potential inducer of oxidative stress [29]. NO_2_^−^ determination was based on the diazotization reaction described by Griess [30]. Briefly, 50 μL of a sulfanilamide solution (1% sulfanilamide diluted in 5% phosphoric acid; Sigma–Aldrich) was added to microplate wells containing 25 μL of the sample. The microplate was incubated for 10 min at room temperature in the dark. Then, a solution of 0.1% N-(1-naphthyl) ethylenediamine dihydrochloride (NED) (Sigma–Aldrich) in milli-Q water was added to the reaction, and samples were read at 540 nm using a microplate reader (VersaMax™, Molecular Devices). The concentration of nitrite in the jejunum was calculated from a standard curve prepared using sodium nitrite (NaNO_2_). The results were expressed as μmol nitrite/mg of protein.

Lipid peroxidation was assessed as described by Buege and Aust [31], on the basis of the ability of thiobarbituric acid to bind to oxidized lipids. Samples were read at 535 nm using the Evolution™ 300 UV-VIS spectrophotometer (Thermo Fisher Scientific™). TBARS content was determined using a molar extinction coefficient of 1.56 × 10^5^ mol/L^.^cm, according to the Beer–Lambert law. Results were expressed as nmol TBARS/mg of protein.

For analysis of protein oxidation, we measured the formation of carbonyl derivatives using the reagent 2,4-dinitrophenylhydrazine (DNPH, Sigma–Aldrich), as described by Levine et al. [32]. Readings were performed at 370 nm on the Evolution™ 300 UV-VIS spectrophotometer (Thermo Fisher Scientific™). Carbonylated protein concentration was determined using the Beer–Lambert equation, *A* = *C × b ×* ε, where *A* is the absorbance of the sample minus that of the control, *C* is the concentration of carbonylated proteins, *b* is the optical path length, and ε is the molar extinction coefficient (22,000 mol/L^.^cm). Results were expressed as nmol carbonylated protein/mg of protein.

Total antioxidant capacity (TAC) was measured by the 2,2-diphenyl-1-picrylhydrazyl (DPPH, Sigma–Aldrich) radical scavenging assay, according to the method described by Brand-Williams et al. [33], with modifications. Briefly, 100 mg of jejunum was added to 1000 μL of methanol. The solution was homogenized using a Van Potter homogenizer and centrifuged at 10,000×*g* for 10 min at 4 °C. Then, 1850 μL of a 6 × 10^− 5^ mol/L DPPH solution was added to an eppendorf tube containing 150 μL of the supernatant obtained from the samples. The solution was kept in the dark for 30 min. Subsequently, samples were read at 515 nm using the Evolution™ 300 UV-VIS spectrophotometer (Thermo Fisher Scientific™). The antioxidant capacity of each sample was calculated as follows: % Antioxidant activity = [1− (absorbance of the sample/absorbance of DPPH)] × 100.

The Bradford method [34] was used to determine total protein content and correct NO_2_^−^, CAT, SOD, carbonylated protein, and TBARS results.

### Gene expression

The jejunum of six birds from each treatment, selected on the basis of the average body weight of each replicate group, was collected 144 h PI, washed with cold sterile saline, and added to an eppendorf tube containing 1 mL of TRIzol™ (Invitrogen, Carlsbad CA, USA). Samples were stored at − 80 °C until total RNA extraction.

Total RNA was extracted according to the manufacturer’s recommendations and quantified spectrophotometrically at 260 nm on a NanoDrop 2000c spectrophotometer (Thermo Fisher Scientific™). RNA integrity was assessed on a 1% agarose gel stained with SYBR™ Safe DNA Gel Stain (Invitrogen, Carlsbad CA, USA) and visualized under ultraviolet light using a photodocumentation system for electrophoresis gel (L-PIX TOUCH, Loccus Biotechnology). To eliminate genomic DNA contamination, we treated 1 μg of total RNA with DNase I, Amplification Grade (Invitrogen, Carlsbad CA, USA), according to the manufacturer’s instructions. Subsequently, complementary DNA (cDNA) synthesis was performed using the SuperScript™ III First-Strand Synthesis SuperMix kit (Invitrogen Corporation, Brazil) according to the manufacturer’s instructions. cDNA concentration was determined at 260 nm using the NanoDrop™ 2000c spectrophotometer (Thermo FisherScientificTM). cDNA samples were stored at − 20 °C until amplification.

RT-qPCR was performed using 5 μL of cDNA diluted to 40 or 80 ng/μL, 0.5 μL or 1 μL of 10 μmol forward primer and 10 μmol reverse primer (final concentrations of 200 nmol and 400 nmol, respectively), 12.5 μL of SYBR™ Green PCR Master Mix (Applied Biosystems™, USA), and UltraPure™ DEPC-treated water (Invitrogen™, Carlsbad CA, USA) to complete the volume to 25 μL. RT-qPCR reactions were performed on a StepOne™ real-time PCR system version 2.3 (Applied Biosystems™), and the thermal cycle for all genes was set to 95 °C for 10 min followed by 40 cycles of denaturation at 95 °C for 15 s and annealing and extension at 60 °C for 1 min, ending with heating from 65 °C to 95 °C for melting curve analysis.

Primers specific to the genes that code for superoxide dismutase 1 (*SOD1*), catalase (*CAT*), peptide transporter 1 (*PEPT1*), neutral and cationic amino acid transporter 1 (*y*^*+*^*LAT-1*), neutral amino acid transporter 1 (*B*^*0*^*AT1*), cationic amino acid transporter 1 (*CAT-1*), toll-like receptor 2 (*TLR2*), toll-like receptor 5 (*TLR5*), interleukin 1 beta (*IL1B*), interleukin 2 (*IL2*), interferon gamma (*IFNG*), lipopolysaccharide-induced tumor necrosis factor (TNF)-alpha factor (*LITAF)*, claudin-1 (*CLDN1*), occludin (*OCLN*), and inducible nitric oxide synthase (*iNOS*) were designed on the basis of gene sequences deposited in the National Center for Biotechnology Information (NCBI, http://www.ncbi.nlm.nih.gov) database (Table 3) using the Integrated DNA Technologies system (http://www.idtdna.com). The β-actin gene (*ACTB*) was used as an endogenous control. All analyses were performed in duplicate, and results were expressed as arbitrary units, AU. The 2^−ΔCT^ method was used to evaluate gene expression [35].

**Table 3:** Primer sequences used for RT-qPCR

Gene	Primer sequences (5′ → 3′)	Amplicon, bp^a^	Accession No.
*SOD1* ^b^	F: AGATGGCAGTGGGAAATGAG	110	NM_205064.1
	R: ACTCAAGACAGCAGAGTAGTAATG		
*CAT*	F: GAGGAACCCTCAGACTCATTTG	117	NM_001031215.2
	R: CCATCAGGAATACCACGATCAC		
*PEPT1*	F: CCCCTGAGGAGGATCACTGTT	66	KF366603.1
	R: CAAAAGAGCAGCAGCAACGA		
*y* ^*+*^ *LAT-1*	F: TGTTGGAGCCAGAGAAGGA	118	XM_418326.6
	R: CACAAGGAGATAAAGCAAAGTC		
*B* ^*0*^ *AT1*	F: TCTATTGAAGATTCGGGCAC	153	XM_419056.6
	R: AATGGTAAGCACAAGGTATGG		
*CAT - 1*	F: CGTGGCATCTCTGCTCATC	134	NM_001145490.1
	R: CTCCATCCCAACCTACATACTTA		
*TLR2*	F: ACTGCCTGCAACGGTCAT	75	NM_204278.1
	R: CATCAGCTTCATTGTTGGTTTCTGT		
*TLR5*	F: ACACGGCAATAGTAGCAACACATAT	91	XM_025148815.1
	R: ACACCTGGAACTTGGAAAAGAACAT		
*IL1B*	F: GTCAACATCGCCACCTACAA	90	XM_015297469.1
	R: CGGTACATACGAGATGGAAACC		
*IL2*	F: CCTCAAGAGTCTTACGGGTCTA	104	GU119890.1
	R: AGTTGGTCAGTTCATGGAGAAA		
*IFNG*	F: TGAGGTGATGTTTACCGAGTTT	94	NM_205149.1
	R: GCTTAGAGCTGAGCAGGTATG		
*LITAF*	F: GAGCGTTGACTTGGCTGTC	64	NM_204267.1
	R: AAGCAACAACCAGCTATGCAC		
*CLDN1*	F: ACTCCTGGGTCTGGTTGGT	100	AY750897.1
	R: GACAGCCATCCGCATCTTCT		
*OCLN*	F: ACGGCAGCACCTACCTCAA	123	D21837.1
	R: GGGCGAAGAAGCAGATGAG		
*iNOS*	F: TCCTGAGTTCTGTGCCTTTG	92	U46504.1
	R: GTTCATCTCCTTCACCCACTG		
*ACTB*	F: GCCAACAGAGAGAAGATGAC	130	L08165.1
	R: CACCAGAGTCCATCACAATAC		

^a^*bp* Base pairs

^b^*SOD1* Superoxide dismutase 1 gene, *CAT* Catalase gene, *PEPT1* Peptide transporter 1 gene, *y*^*+*^*LAT-1* Neutral and cationic amino acid transporter 1 gene, *B*^*0*^*AT1* System B^0^ neutral amino acid transporter 1 gene, *CAT–1* Cationic amino acid transporter 1 gene, *TLR2* Toll-like receptor 2 gene, *TLR5* Toll-like receptor 5 gene, *IL1B* Interleukin 1 beta gene, *IL2* Interleukin 2 gene, *IFNG* Interferon gamma gene, *LITAF* Lipopolysaccharide-induced tumor necrosis factor-alpha factor gene, *CLDN1* Claudin-1 gene, *OCLN* Occludin gene, *iNOS* Inducible nitric oxide synthase gene, *F* Forward, *R* Reverse

### Statistical analysis

The Shapiro–Wilk test was applied to verify the normality of data distribution. Data were analyzed by two-way ANOVA, which considers the main effects (*Eimeria* spp*.* challenge and methionine supplementation) and the interaction between the factors. Means were compared by the Tukey test (*P* < 0.05) using SAS 2002 version 9.00 (SAS Inst. Inc., Cary, NC).

## Results

**Fig. 1:**
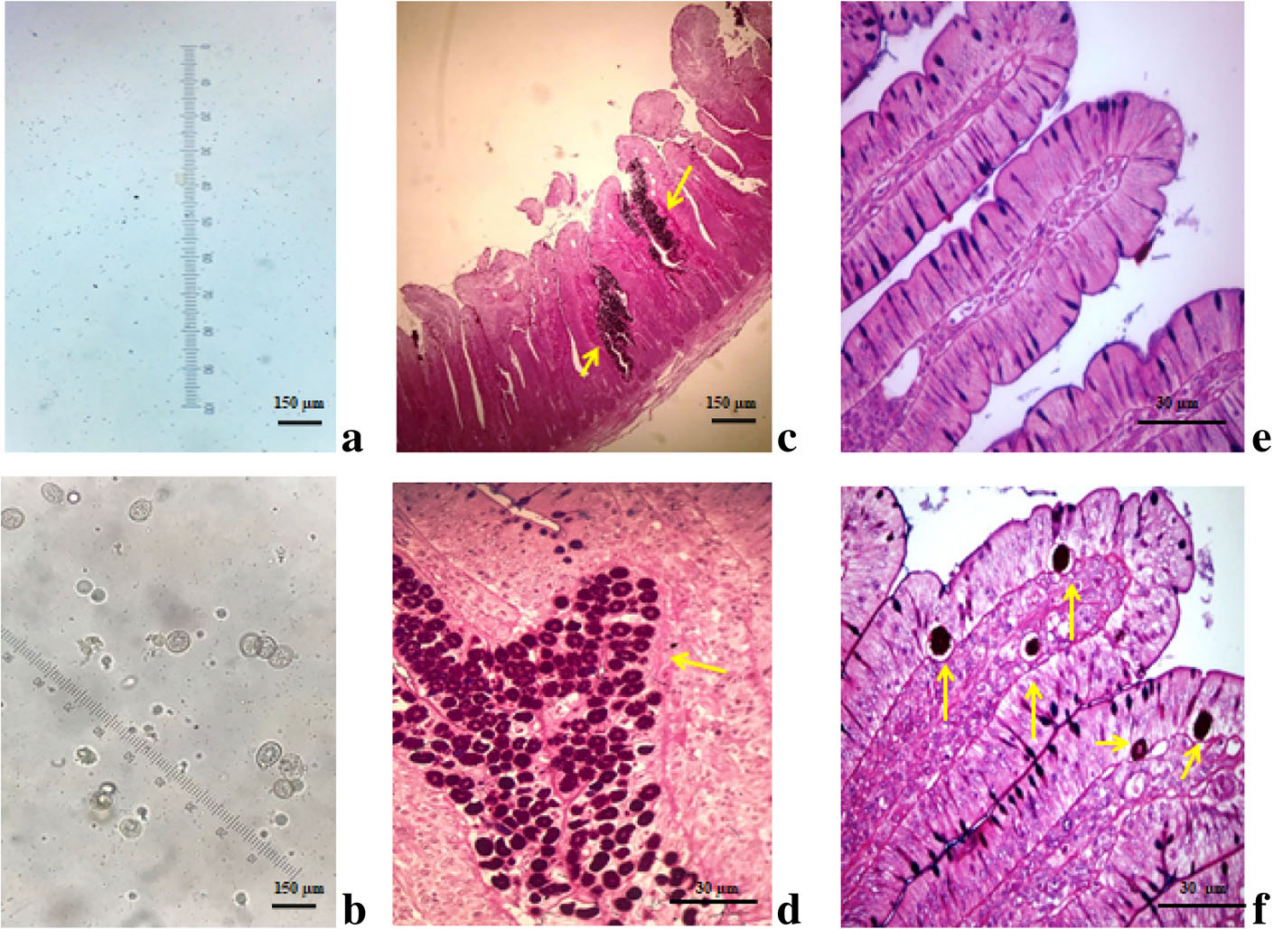
Oocysts detection in the feces of unchallenged, UC **a** and *Eimeria*-challenged, EC **b** broiler chickens 144 h post-inoculation (PI). **c**, **d** Histological images of duodenal villi and **e**, **f** jejunal villi in broiler chickens 144 h PI, 40× magnification. Note the presence of *Eimeria* structures (yellow arrows) in the duodenal and jejunal mucosa of EC broilers. **e** Intact jejunal villi in UC broilers. Hematoxylin-eosin staining. **a**, **b**, **c** Scale bars represent 150 μm. **d**, **e**, **f** Scale bars represent 30 μm

The absence of oocysts in the excreta of UC broilers validates their use as controls (Fig. 1a). The infection caused by *Eimeria* spp. in EC broilers was confirmed by the presence of oocysts in the excreta samples (Fig. 1b), histological alterations in the intestinal mucosal (Fig. 1c), and the presence of *Eimeria* spp. structures in duodenal enterocytes (Fig. 1d) and in jejunal enterocytes and lamina propria (Fig. 1f). The jejunal villi of UC broilers were normal, confirming the absence of infection and the integrity of the intestinal epithelium (Fig. 1e).

### Animal performance and relative weight of organs

*Eimeria* spp*.* challenge and methionine supplementation showed no interaction effect on performance parameters (Table 4). However, FI, BWG, and FCR values of EC chickens were respectively 13% lower, 37% lower, and 39% higher (*P* < 0.0001) than those of UC chickens. Methionine supplementation had a significant effect on BWG (*P* = 0.0038) and FCR (*P* = 0.0190), as chickens fed the dl-Met diet had higher BWG (about 12% higher) and better FCR (about 12% lower) than chickens fed the NS diet. No differences were observed between chickens fed dl-Met and dl-MMet diets.

**Table 4:** Performance of broilers 144 h post-inoculation with *Eimeria* spp.

		FI, kg^c^		BWG, kg^d^		FCR^e^	
		Mean	SE	Mean	SE	Mean	SE
UC^f^	NS^h^	0.470	0.011	0.295	0.009	1.593	0.016
	*dl*-Met^i^	0.498	0.009	0.333	0.006	1.515	0.042
	*dl*-MMet^j^	0.493	0.015	0.315	0.015	1.568	0.115
EC^g^	NS	0.428	0.006	0.183	0.009	2.378	0.108
	dl-Met	0.420	0.009	0.213	0.003	1.987	0.047
	dl-MMet	0.415	0.006	0.198	0.005	2.101	0.069
Main effects							
Challenge	UC	0.487^a^	0.007	0.314^a^	0.007	1.558^b^	0.038
	EC	0.421^b^	0.004	0.198^b^	0.005	2.155^a^	0.064
Diet	NS	0.449	0.010	0.239^b^	0.022	1.985^a^	0.157
	*dl*-Met	0.459	0.016	0.273^a^	0.023	1.751^b^	0.094
	*dl*-MMet	0.454	0.016	0.256^ab^	0.023	1.834^ab^	0.118
*P*-value							
Challenge		< 0.0001		< 0.0001		< 0.0001	
Diet		0.6033		0.0038		0.0190	
Challenge × Diet		0.1487		0.9062		0.1155	

^a,b^Means in the same column followed by different letters differ significantly by the Tukey test, *P* < 0.05. Results are presented as mean ± standard error, SE. Each cage was considered an experimental unit, *n* = 8. No mortality was observed during the experimental period

^c^*FI* Feed intake. Performance data were calculated taking into consideration a period of 6 days, that is, the period of 144 h post-inoculation, PI with *Eimeria* spp.

^d^*BWG* Body weight gain. Performance data were calculated taking into consideration a period of 6 days, that is, the period of 144 h post-inoculation, PI with *Eimeria* spp.

^e^*FCR* Feed conversion ratio. Performance data were calculated taking into consideration a period of 6 days, that is, the period of 144 h post-inoculation, PI with *Eimeria* spp.

^f^*UC* Unchallenged broilers

^g^*EC Eimeria-*challenged broilers

^h^*NS* non-supplemented diet

^i^*dl-Met* diet supplemented with free methionine, *dl*-methionine 99%

^j^*dl-MMet* diet supplemented with methionine dipeptide, *dl*-methionyl-*dl*-methionine 95%

**Fig. 2:**
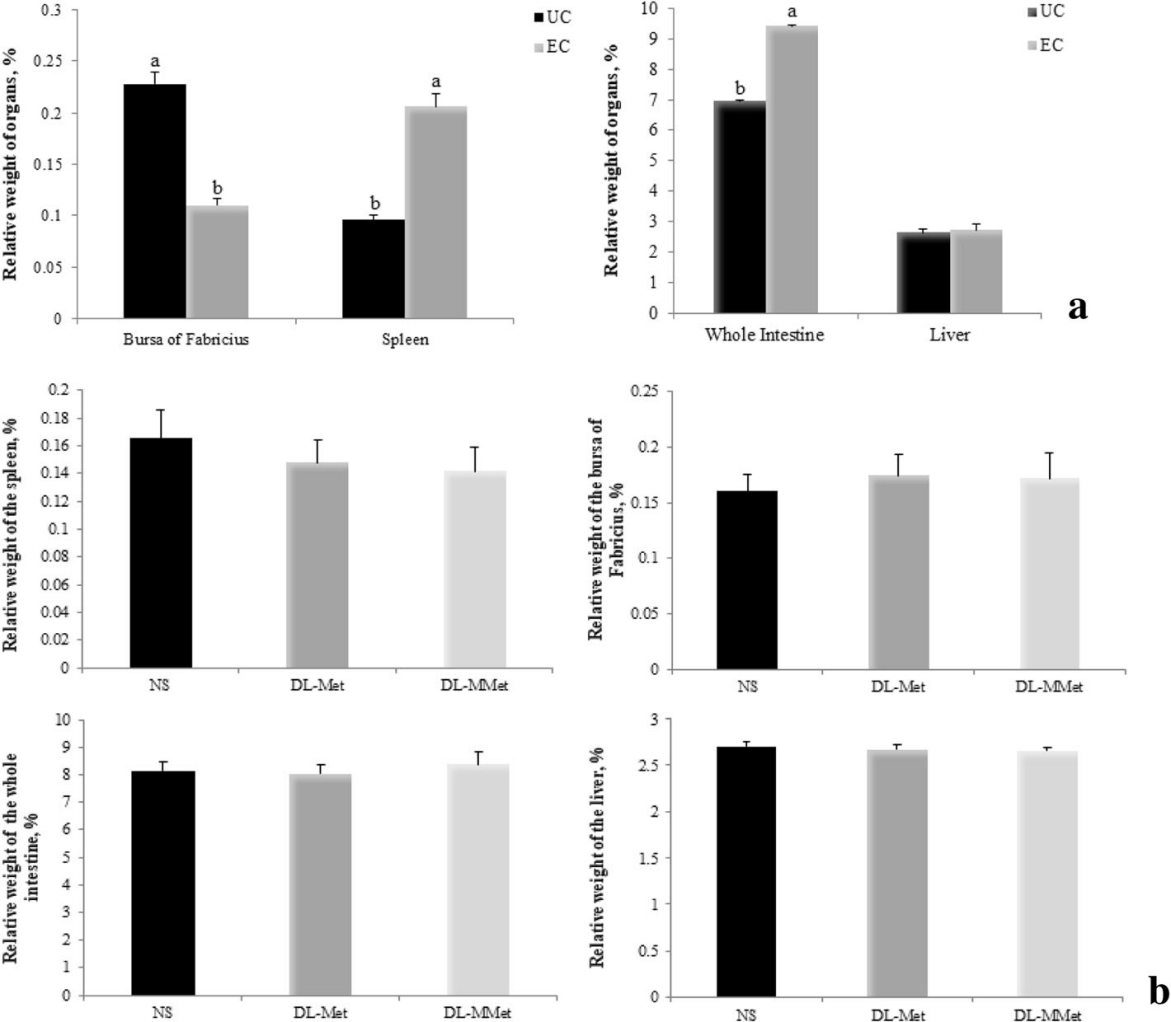
Effects of *Eimeria* spp. challenge **a** and methionine supplementation **b** on the relative weight of organs of broilers. Results are presented as mean and standard error. ^a,b^ Different letters represent significant differences between treatments by the Tukey test, *P* < 0.05. UC = unchallenged broilers; EC = *Eimeria-*challenged broilers; NS = non-supplemented diet; *dl*-Met = diet supplemented with free methionine; *dl*-MMet = diet supplemented with methionine dipeptide

The effects of *Eimeria* spp*.* challenge and methionine supplementation on the relative weight of organs are presented in Fig. 2a and Fig. 2b, respectively. *Eimeria* spp. challenge had significant effects on the relative weight of the bursa of Fabricius (*P* < 0.0001), spleen (*P* < 0.0001), and whole intestine (*P* < 0.0001) (Fig. 2a). EC birds had lower relative weight of the bursa of Fabricius and higher relative weight of the spleen and whole intestine than UC birds (Fig. 2a). There was no effect of *Eimeria* spp*.* challenge on liver weight (Fig. 2a). Methionine supplementation had no effect on the relative weight of the organs (*P* > 0.05) (Fig. 2b).

### Redox state of the jejunum

An interaction effect between *Eimeria* spp*.* challenge and methionine supplementation on TAC, CP, and CAT and SOD activities in the jejunum of chickens was observed (Table 5). Within the UC group, there was no effect of diet on CP; however, chickens fed the *dl*-Met diet had higher TAC and lower CAT and SOD activities. Regarding EC chickens, the highest level of carbonylated protein (CP) was found in the jejunum of chickens fed the NS diet. This result was associated with lower TAC and CAT activity. EC chickens fed the *dl*-Met and *dl*-MMet diets had CP levels similar to those of UC chickens fed the same diets, indicating the role of methionine as an antioxidant in the metabolism of EC chickens.

**Table 5:** Carbonylated protein, CP, total antioxidant capacity, TAC, and antioxidant enzyme activities in the jejunum of broilers 144 h post-inoculation with *Eimeria* spp

		CP^d^		TAC^e^		Catalase^f^		SOD^g^	
		Mean	SE	Mean	SE	Mean	SE	Mean	SE
UC^h^	NS^j^	1.05^bc^	0.11	64.36^c^	3.52	28.27^a^	2.06	4.49^ab^	0.09
	*dl*-Met^k^	1.04^bc^	0.17	81.89^a^	2.15	21.47^bc^	1.42	3.82^c^	0.04
	*dl*-MMet^l^	1.27^ab^	0.16	71.39^bc^	1.69	27.78^a^	1.28	3.69^c^	0.13
EC^i^	NS	1.43^a^	0.10	65.27^c^	2.10	19.62^c^	1.25	4.72^a^	0.05
	*dl*-Met	0.85^c^	0.15	67.45^c^	3.06	24.77^ab^	1.94	4.32^b^	0.11
	*dl*-MMet	0.90^bc^	0.07	78.32^ab^	2.52	22.38^bc^	0.49	3.59^c^	0.14
*P*-value									
Challenge × Diet		0.0200		0.0008		0.0012		0.0214	

^a,b,c^Means in the same column followed by different letters differ significantly by the Tukey test, *P* < 0.05. Results are presented as mean ± standard error, SE. Each bird was considered an experimental unit, *n* = 6

^d^Expressed as nmol of carbonyl groups/mg of protein

^e^TAC = total antioxidant capacity, expressed as percentage, %

^f^Catalase activity, expressed as the amount of hydrogen peroxide, H_2_O_2_ consumed per minute per milligram of protein

^g^SOD = superoxide dismutase activity, expressed as SOD unit/mg of protein

^h^UC = unchallenged broilers

^i^EC = *Eimeria-*challenged broilers

^j^NS = non-supplemented diet

^k^*dl*-Met = diet supplemented with free methionine (*dl*-methionine 99%)

^l^*dl*-MMet = diet supplemented with methionine dipeptide (*dl*-methionyl-*dl*-methionine 95%)

**Fig. 3:**
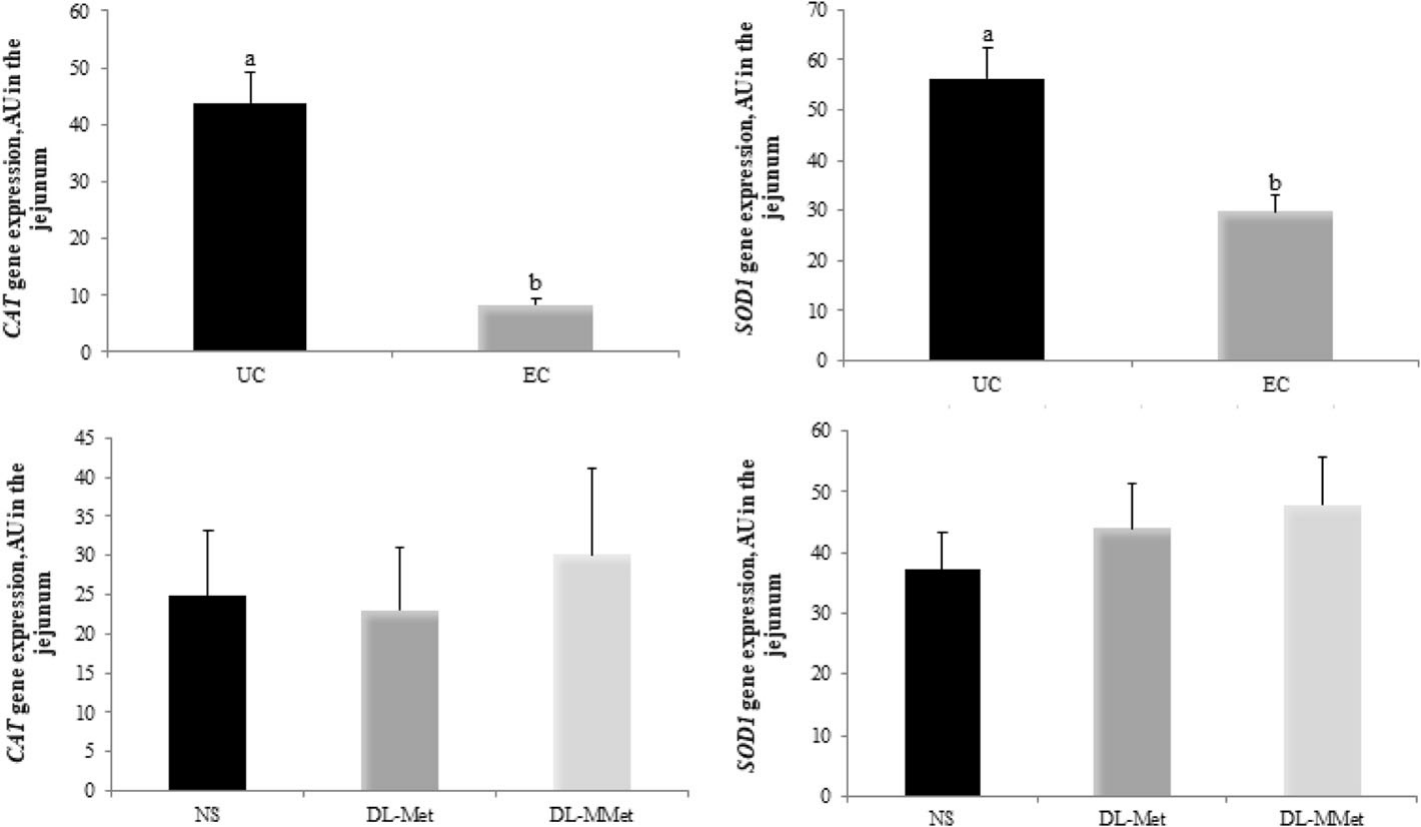
Expression of catalase, *CAT* and superoxide dismutase 1, *SOD1* genes in the jejunum of broiler chickens. Results are expressed as arbitrary units, AU, and are presented as mean and standard error. ^a,b^ Different letters represent significant differences between treatments by the Tukey test, *P* < 0.05. UC = unchallenged broilers; EC = *Eimeria-*challenged broilers. NS = non-supplemented diet; *dl*-Met = diet supplemented with free methionine; *dl*-MMet = diet supplemented with methionine dipeptide

The lower TAC observed in EC chickens might also be associated with the reduced expression of *CAT* (*P* < 0.0001) and *SOD1* (*P* = 0.0036) genes (Fig. 3). There was no effect of methionine supplementation on the expression of these genes.

**Fig. 4:**
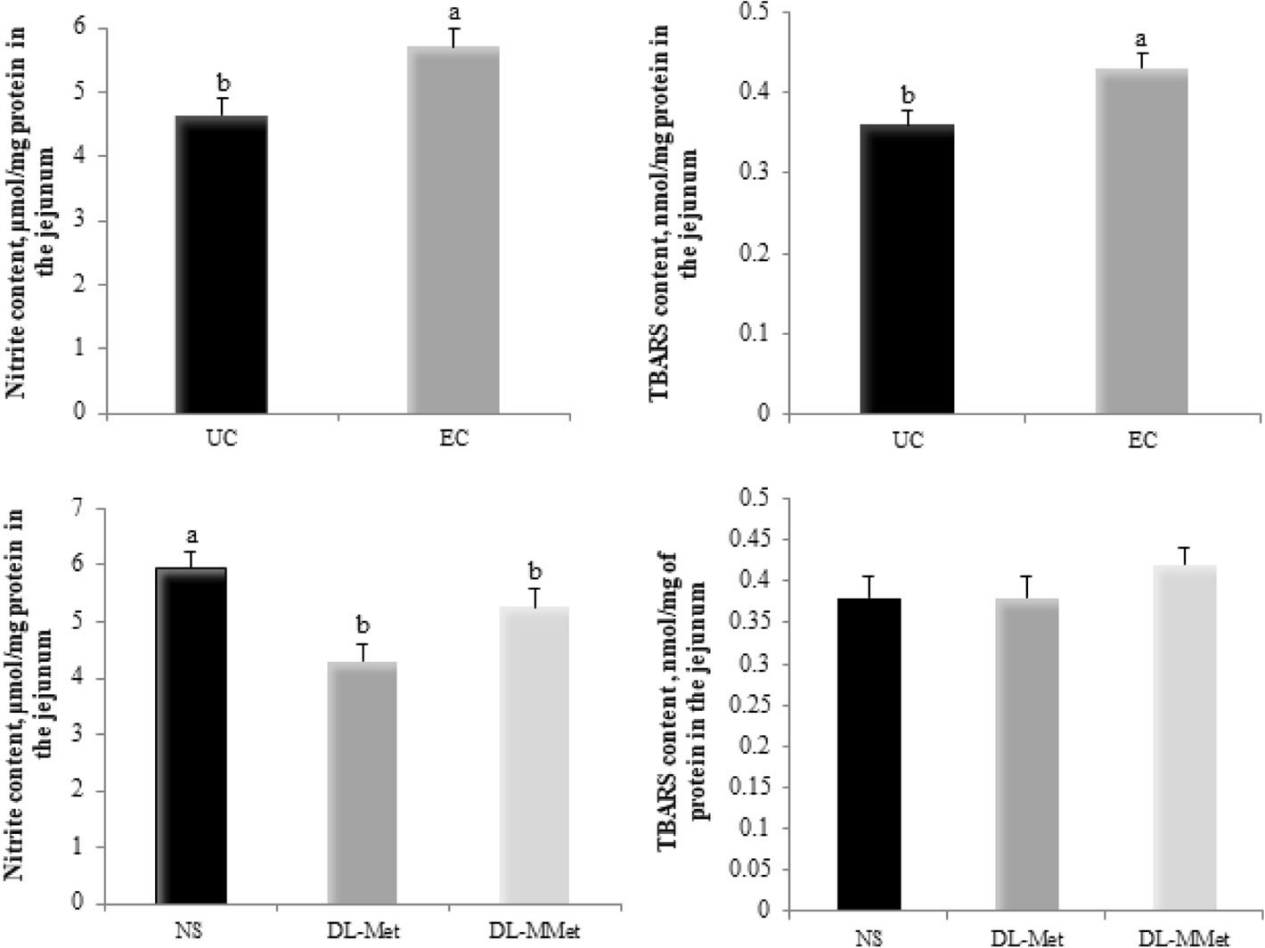
Effect of *Eimeria* spp. challenge and methionine supplementation on nitrite, NO_2_^−^ content and thiobarbituric acid reactive substances, TBARS content in the jejunum of broiler chickens 144 h post-inoculation. Results are presented as mean and standard error. ^a,b^ Different letters represent significant differences by the Tukey test, *P* < 0.05. UC = unchallenged broilers; EC = *Eimeria-*challenged broilers; NS = non-supplemented diet; *dl*-Met = diet supplemented with free methionine; *dl*-MMet = diet supplemented with methionine dipeptide

EC chickens had higher nitrite and TBARS contents than UC chickens. Birds fed the *dl*-Met and *dl*-MMet diets showed lower nitrite contents than birds fed the NS diet. There was no effect of methionine supplementation on TBARS content (Fig. 4).

### Expression of genes associated with intestinal function and integrity

**Fig. 5:**
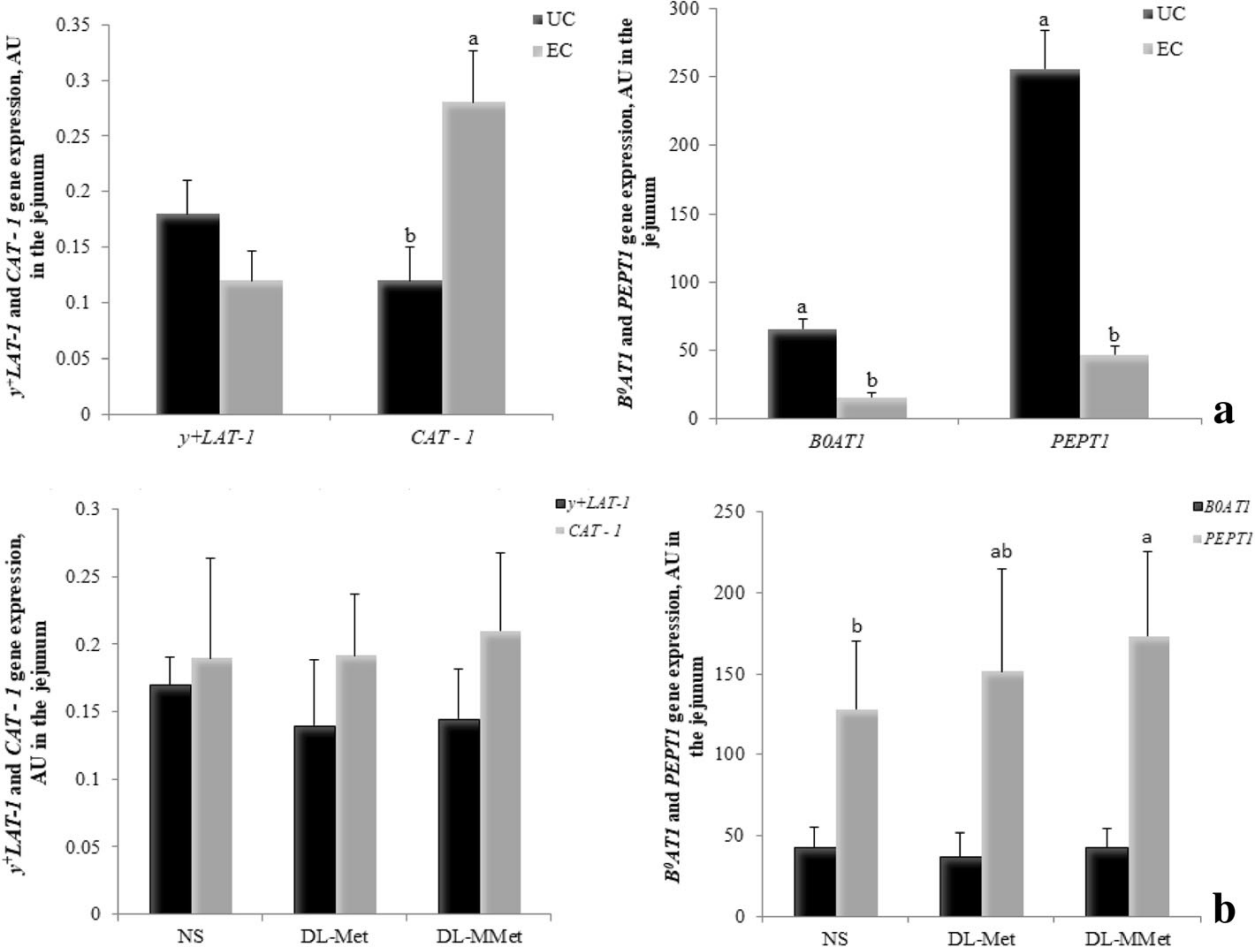
Effect of *Eimeria* spp. challenge **a** and methionine supplementation **b** on the expression of genes encoding *y*^*+*^*LAT-1, B*^*0*^*AT1, CAT-1,* and *PEPT1* in the jejunum of broilers. Results are expressed as arbitrary units, AU, and are presented as mean and standard error. ^a,b^ Different letters represent significant differences between treatments by the Tukey test, *P* < 0.05. UC = unchallenged broilers; EC = *Eimeria-*challenged broilers. NS = non-supplemented diet; *dl*-Met = diet supplemented with free methionine; *dl*-MMet = diet supplemented with methionine dipeptide. *y*^*+*^*LAT-1* = neutral and cationic amino acid transporter 1 gene. *CAT-1* = cationic amino acid transporter 1 gene; *B*^*0*^*AT1* = system B^0^ neutral amino acid transporter 1 gene; *PEPT1* = peptide transporter 1 gene

We investigated the effects of the treatments on the expression of genes related to the function and integrity of the intestinal tract. The expression of genes encoding *y*^*+*^*LAT-1*, *B*^*0*^*AT1, CAT-1,* and *PEPT1* in the jejunum of broilers 144 h PI is shown in Fig. 5a and Fig. 5b. *Eimeria* spp. challenge affected the expression of *B*^*0*^*AT1* (*P* = 0.0001), *CAT-1* (*P* = 0.0300), and *PEPT1* (*P* < 0.0001) (Fig. 5a). EC chickens showed lower *B*^*0*^*AT1* (0.1589 AU vs. 0.2166 AU) and *PEPT1* (0.4644 AU vs. 2.5611 AU) and higher *CAT - 1* (0.0028 AU vs. 0.0012 AU) gene expression than UC chickens. *Eimeria* spp. challenge had no significant effect on *y*^*+*^*LAT-1* expression (*P* > 0.05) (Fig. 5a). Methionine supplementation (Fig. 5b) influenced *PEPT1* expression (*P* = 0.0467): broilers fed the dl-MMet diet had higher expression levels of this gene. There was no effect of methionine supplementation on the expression of the genes *y*^*+*^*LAT-1, CAT-1*, and *B*^*0*^*AT1* (*P* > 0.05) (Fig. 5b).

**Fig. 6:**
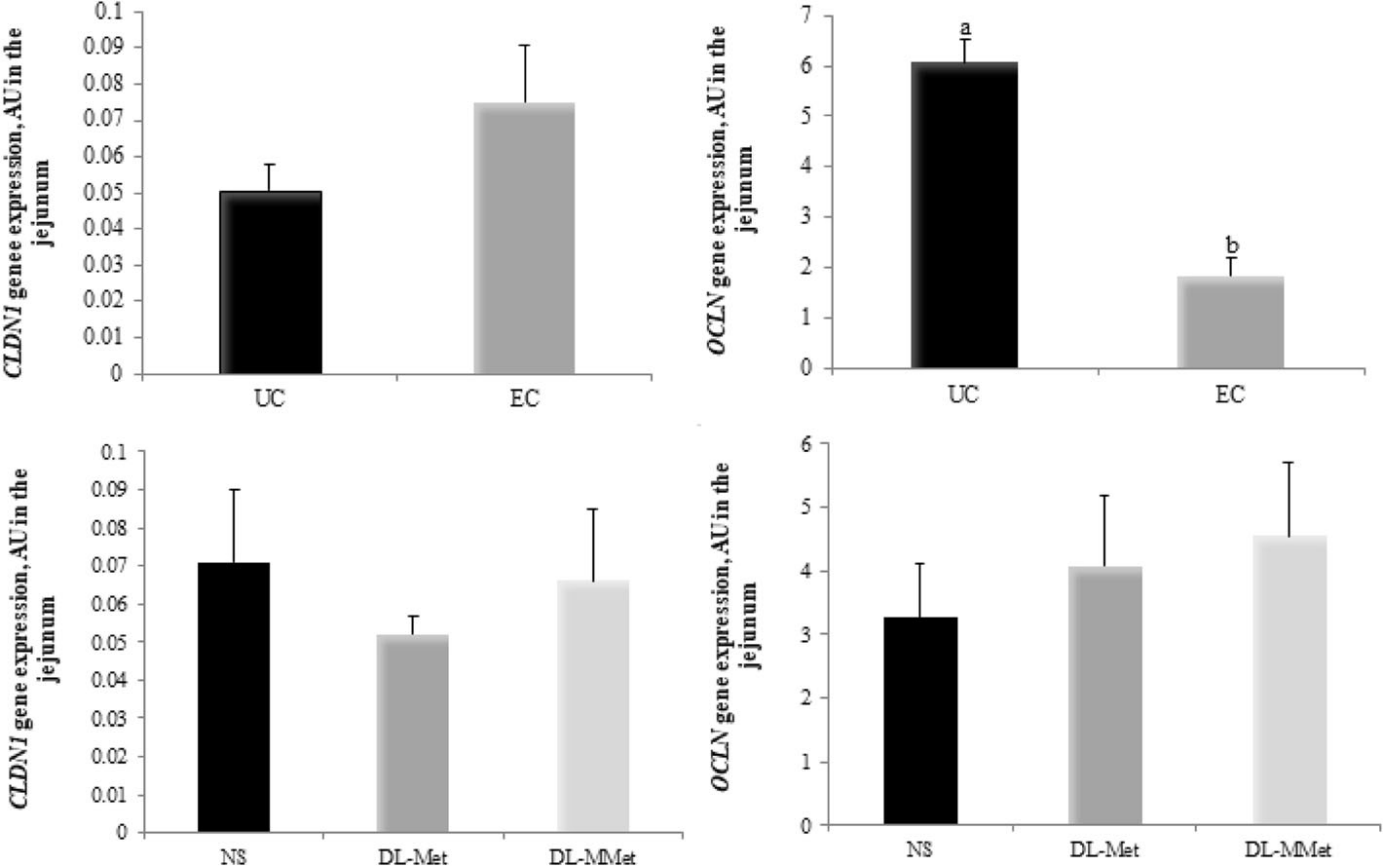
Effect of *Eimeria* spp. challenge and methionine supplementation on the expression of genes encoding claudin-1, *CLDN1* and occludin, *OCLN* in the jejunum of broiler chickens 144 h post-inoculation. Results are expressed as arbitrary units, AU, and are presented as mean and standard error. ^a,b^ Different letters represent significant differences between treatments by the Tukey test, *P* < 0.05. NS = non-supplemented diet; *dl*-Met = diet supplemented with free methionine; *dl*-MMet = diet supplemented with methionine dipeptide

Figure 6 shows the results of *CLDN1* and *OCLN* gene expression*.* EC chickens had decreased expression of *OCLN* compared to UC chickens. There was no effect of *Eimeria* spp. challenge on *CLDN1* expression (*P* = 0.1825), and no effect of methionine supplementation on the expression of *CLDN1* (*P* = 0.6416) and *OCLN* (*P* = 0.1595).

### Expression of genes associated with immune responses

**Fig. 7:**
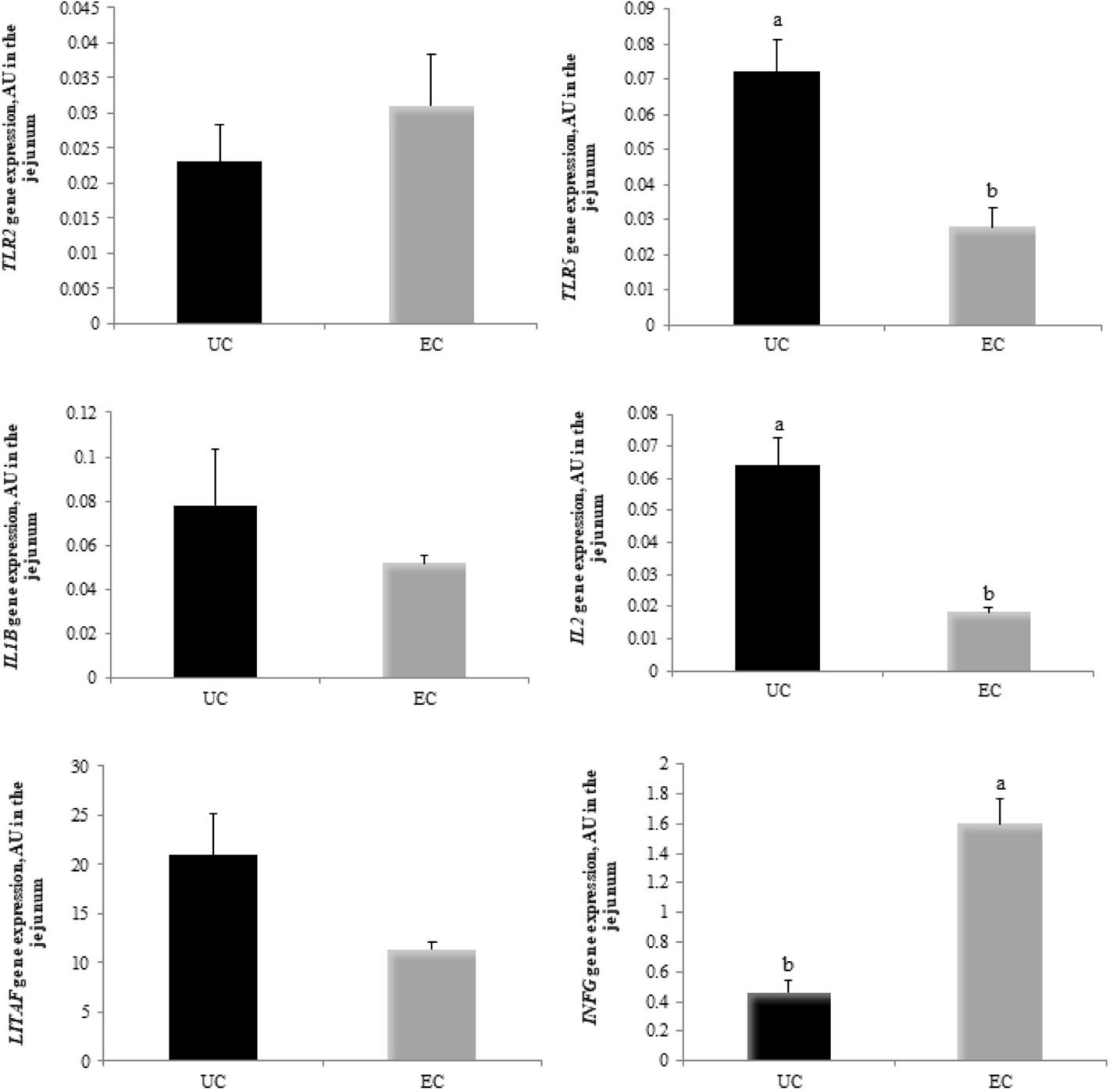
Expression of genes associated with the immune system in the jejunum of broilers 144 h post-inoculation with *Eimeria* spp. Results are expressed as arbitrary units, AU, and are presented as mean and standard error. ^a,b^ Different letters represent significant differences between treatments by the Tukey test, *P* < 0.05. UC = unchallenged broilers; EC = *Eimeria-*challenged broilers. *TLR2* = toll-like receptor 2 gene; *TLR5* = toll-like receptor 5 gene; *IL1B* = interleukin 1 beta gene; *IL2* = interleukin 2 gene; *LITAF* = lipopolysaccharide-induced tumor necrosis factor-alpha factor gene; *IFNG* = interferon gamma gene

The effects of *Eimeria* spp. challenge on the relative expression of *TLR2, TLR5, IL1B, IL2, LITAF,* and *IFNG* genes are shown in Fig. 7. *Eimeria* spp*.* challenge significantly reduced the expression of *TLR5* (*P* = 0.0003) and *IL2* (*P* = 0.0001) and significantly increased the expression of *IFNG* (*P* = 0.0001). However, no significant effect of *Eimeria* spp*.* challenge on the expression of *TLR2*, *IL1B*, and *LITAF* was observed (*P* > 0.05) (Fig. 7).

**Fig. 8:**
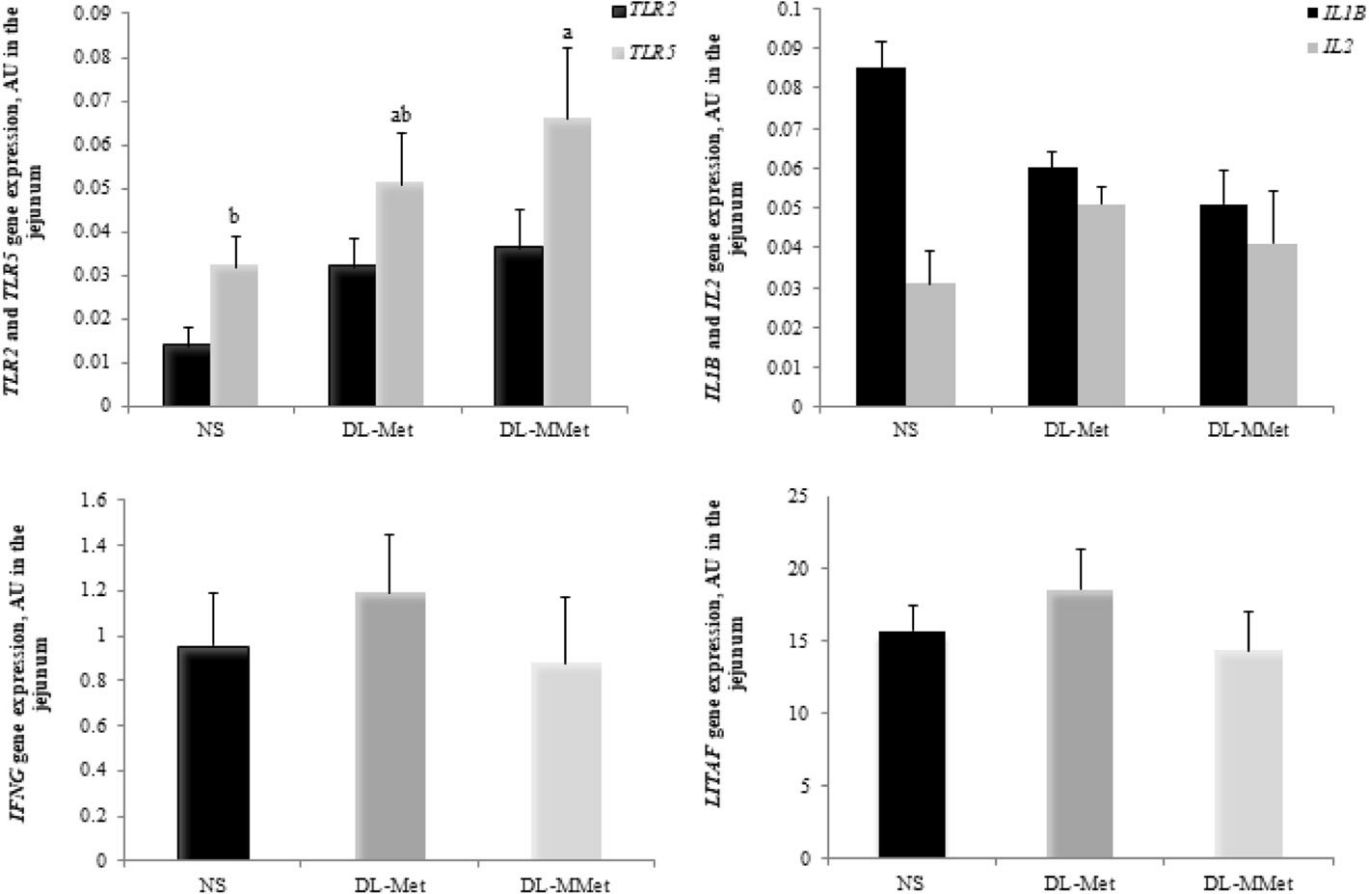
Effect of methionine supplementation on the expression of the toll-like receptor 2 gene, *TLR2*, toll-like receptor 5 gene, *TLR5*, interleukin 1 beta gene, *IL1B*, interleukin 2 gene, *IL2*, interferon gamma gene, *IFNG*, and lipopolysaccharide-induced tumor necrosis factor-alpha factor, *LITAF* gene in the jejunum of broiler chickens. Results are expressed as arbitrary units, AU, and are presented as mean and standard error. ^a,b^ Different letters represent significant differences between treatments by the Tukey test, *P* < 0.05. NS = non-supplemented diet; *dl*-Met = diet supplemented with free methionine; *dl*-MMet = diet supplemented with methionine dipeptide

The effects of diet on the relative expression of *TLR2, TLR5, IL1B, IL2, IFNG*, and *LITAF* genes are presented in Fig. 8. Chickens fed the NS diet had lower expression of *TLR5* than chickens fed the *dl*-MMet diet (*P* = 0.0255). There was no difference between the sources of methionine. No significant effect was observed on the expression of *TLR2, IL1B,* and *LITAF* (*P* > 0.05) (Fig. 8).

**Fig. 9:**
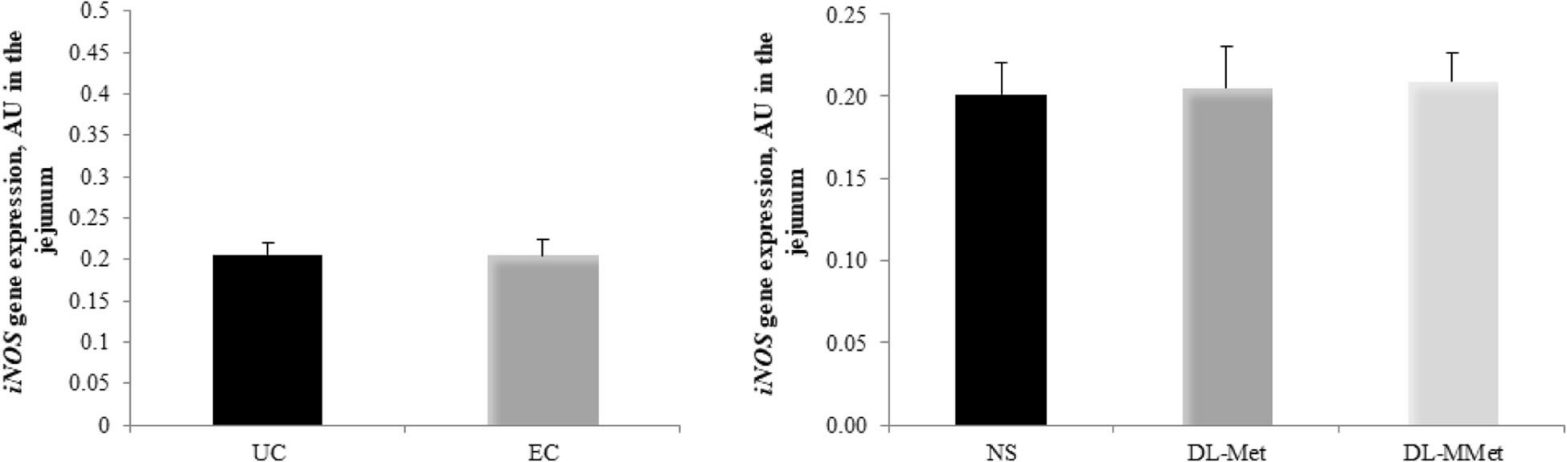
Effect of *Eimeria* spp. challenge and methionine supplementation on the expression of inducible nitric oxide synthase gene, *iNOS* in the jejunum of broilers 144 h post-inoculation. Results are expressed as arbitrary units, AU, and are presented as mean and standard error. UC = unchallenged broilers; EC = *Eimeria-*challenged broilers. NS = non-supplemented diet; *dl*-Met = diet supplemented with free methionine; *dl*-MMet = diet supplemented with methionine dipeptide

No effect of *Eimeria* spp. challenge or methionine supplementation was observed on the expression of the gene *iNOS* (Fig. 9) (*P* > 0.05).

## Discussion

Previous studies showed that broilers challenged with *Eimeria* spp. have poor performance in part because of structural and functional changes that occur in the intestinal mucosa during the infection [5, 7, 8]. We observed that EC chickens had FI and BWG 13% and 37% lower than UC chickens, respectively. The decreased BWG in EC chickens was followed by an increase of 39% in FCR. The reduced FI in EC chickens might be related, among other factors, to the increase in plasma cholecystokinin secretion, a potent inhibitor of feeding [36]. Low BWG may have been caused by several factors, such as low FI and low expression of the genes *B*^*0*^*AT1* and *PEPT1* in the jejunum. *B*^*0*^*AT1* encodes the neutral amino acid transporter (B^0^AT1), and *PEPT1* encodes a protein responsible for the uptake of di- and tripeptides. Thus, a reduced expression of *B*^*0*^*AT1* and *PEPT1* genes could be associated with decreased absorption of amino acids and decreased protein synthesis.

PepT 1 is highly resilient to changes in the gut caused by intestinal damage [18, 21]. We hypothesized that dl-MMet supplementation could exert a beneficial effect on animal metabolism during *Eimeria* spp. challenge. No interaction effect between *Eimeria* spp. challenge and methionine supplementation on the expression of *PEPT1* was observed. However, there was a significant reduction in *PEPT1* expression in the jejunum of EC broilers compared with that of UC broilers and a higher expression in animals fed the *dl*-Met and *dl*-MMet diets than in those fed the NS diet. The lower *PEPT1* expression in EC birds might be due to lower nutrient intake, as they exhibited lower FI. Chen et al. [37] suggest that end products of protein digestion (small peptides and amino acids) may be involved in *PEPT1* expression pathways. We suggest that methionine stimulates *PEPT1* expression and that, because PepT 1 is a low-affinity, high-capacity transporter, increased expression of these gene could be related to higher nutrient absorption [18]. Furthermore, we found that chickens fed diets with methionine supplementation had higher BWG and better FCR than chickens fed the NS diet. Although studies indicate that, in some species, dipeptides are more readily absorbed, and thus more readily available, than free amino acids [17, 21, 22] in this study we did not observe differences in the BWG of animals fed *dl*-Met or *dl*-MMet diets. This result may be related to the bioefficacy of methionine sources. Similar bioefficacy values were observed between chickens fed free methionine (*dl*-M) and methionine dipepitde (*dl*-MM) diets in a study by Silva et al. [38]. A thorough investigation on the nutritional and metabolic effects of dipeptide methionine supplementation for chickens is needed.

The effects of coccidiosis are not limited to poor animal performance. Several changes in the composition of organs and tissues were observed in animals infected with coccidia [39]. As the weight of lymphoid organs can be used as an indication of the body’s ability to deliver lymphoid cells during immune responses [40], we evaluated the relative weight of the bursa of Fabricius and of the spleen. We observed that EC broilers showed lower relative weight of the bursa of Fabricius, as reported in other study [41], and higher relative weight of the spleen and whole intestine, which is also in agreement with the results of previous works [42, 43]. A low relative weight of the bursa of Fabricius can be attributed to organ atrophy caused by lymphocyte depletion or to micronecrosis and cell migration [44]. Increased relative weight of the spleen can represent the body’s attempt to meet the requirements of the humoral and cellular immunity [42]. Increased intestine weight might be associated with edema formation, thickening of the mucosa [1], hypertrophy, or mucosal hyperplasia as a result of cell proliferation to replace damaged epithelial cells [45].

During the course of the infection, immunological reactions generate a microenvironment that is incompatible with the survival of pathogens and confer protective immunity against *Eimeria* spp*.* reinfection [1]. The immune system of a parasitized animal detects and responds rapidly to infection through innate immune receptors [46], such as the transmembrane TLRs, which play an important role in immune responses against *Eimeria* spp. [47]. TLRs are expressed by most immune cells [48] as well as by intestinal epithelial cells (IECs) and lamina propria cells [49]. Recognition of pathogen-associated molecular patterns (PAMPs) triggers TLR signaling via myeloid differentiation primary response 88 (MyD88) and TIR-domain-containing adapter-inducing interferon-β (TRIF), inducing the expression of IL-12, IFNs, and TNF [50], which are important effector molecules in innate and adaptive immunity against pathogenic microorganisms [50, 51]. EC chickens showed lower *TLR5* expression than UC chickens. This result might be related to the time of sample collection (144 h PI). According to Netea et al. [52], the innate immune system is activated within minutes of invasion by the protozoan and remains active mainly during the first days of infection. On the other hand, chickens fed the NS diet had the lowest *TLR5* expression levels. Zhang et al. [50] suggested that high expression of *TLR5* can be caused by TLR5 recognition of flagellin in the normal intestinal flora of animals. According to Tang et al. [53], methionine influences the intestinal microflora by stimulating the growth of beneficial bacteria. Our results indicate that methionine deficiency negatively affects immune response activation, possibly because it causes changes in the intestinal flora normal of chickens. EC chickens showed lower *IL2* expression than UC chickens. According to Kaiser et al. [54], low IL2 levels can result in slower inflammatory responses in the intestine, allowing invasion of intestinal epithelial cells by *Eimeria* spp. and progression of the parasite life cycle. Our results may also be associated with the higher expression of the interleukin 10 (*IL10*) gene, which is generally observed in *Eimeria*-challenged chickens [51], as IL10 can inhibit the synthesis of cytokines including IL1β, IL2, and TNF-α [55]. Sand et al. [56] suggested that the potentially inappropriate activation of IL10 is a defense strategy used by *Eimeria* spp. to escape immune detection in the intestinal tract of the host. Interferon gamma plays a critical role in mediating protective immunity against coccidiosis [57]. EC chickens had higher *IFNG* expression than UC chickens, a finding that can be explained by the importance of this cytokine as a mediator of resistance to protozoans, including those of the genus *Eimeria* [51]. Studies demonstrated that interferon gamma levels increase during coccidiosis and that this molecule plays a particular role in the activation of intracellular toxicity via free radical generation and decreasing oocyst production during primary infection with *Eimeria* spp. [57, 58]. However, the inflammatory process in the intestine of a parasitized animal is accompanied by damages to the intestinal epithelium and tight junctions (TJs). Claudins and occludin are dynamic TJ proteins that function as a selective/semipermeable barrier that facilitates the transport of ions and solutes through the paracellular space while preventing the passage of luminal antigens, microorganisms, and toxins [59]. Chickens with coccidiosis have lower occludin gene expression levels as a consequence of multiple factors, including inflammation [60]. We suggest the low *OCLN* expression observed in this study might be due to the immune responses triggered by interferon gamma [61]. Low occludin levels are associated with the disruption of TJs in the jejunum and consequently with the passive loss of solutes and the osmotically driven flow of water into the intestine, resulting in diarrhea, one of the main signs of intestinal inflammation [60].

Interferon gamma also affects the intracellular replication of *Eimeria* spp. [57] because it is a potent activator of inducible nitric oxide synthase (iNOS), an enzyme responsible for the production of nitric oxide (NO), which in turn has been proposed to be the effector molecule against *Eimeria* spp. [62]. NO is a highly reactive free radical synthesized from l-arginine by nitric oxide synthases (NOS) [63, 64]. This free radical performs a variety of biological functions [65] and acts as a cytotoxic agent against tumor cells, bacteria, viruses, and parasites [64]. Because of its oxidizing effect and ability to react with intracellular compounds, NO is generated in large quantities during immunological reactions [66].

In this study, we quantified the nitrite content in the jejunum of EC and UC chickens and evaluated both *iNOS* and *CAT-1* expression. The *CAT-1* gene codes for CAT-1, a protein involved in the absorption of arginine, which is the substrate for NO synthesis. *Eimeria* spp*.* infection had no effect on *iNOS* expression but increased *CAT-1* expression and nitrite content. The lack of significant results for *iNOS* expression can be due to the time of sampling (144 h PI). Once expressed, *iNOS* produces high levels of NO for periods that can last from hours to days [63]. Overall, these results suggest that a highly complex and integrated intestinal defense mechanism is activated in an attempt to fight the coccidial infection via increased expression of *IFNG* and *CAT-1* and, consequently, enhanced production of NO_2_^−^, which is toxic for coccidians [62].

Studies evidenced the occurrence of oxidative stress in chickens challenged with *Eimeria* spp. [2, 9, 10]. Oxidative imbalance in chickens with coccidiosis is caused by reduced antioxidant enzyme activity; reduced levels of non-enzymatic antioxidants, such as carotenoids, vitamin C, and vitamin E; increased lipid peroxidation; and increased NO_2_^−^ and nitrate (NO_3_^−^) levels [2, 9–11]. In the present study, we found that EC chickens 144 h PI had higher TBARS and nitrite contents in the jejunum than UC chickens. These alterations may be due to the effects of NO and oxygen-derived radicals, which produce other toxic substances, such as the potent oxidizing agent peroxynitrite [67], and result in lipid peroxidation. Oxidative stress also causes damage to others biological molecules. Proteins are possibly the most immediate vehicle for inflicting oxidative damage on cells because they usually act as catalysts rather than as stoichiometric mediators [68]. Reactions between proteins and free radicals can lead to the formation of protein derivatives or peptide fragments that possess highly reactive carbonyl groups. In fact, the accumulation of carbonylated proteins has been observed in several diseases, including intestinal diseases [69, 70]. EC chickens fed the NS diet showed high carbonylated protein content, indicating that peroxynitrite formation might have occurred during the infection, causing severe oxidative damage to proteins. Moreover, this result shows that methionine deficiency enhances oxidative stress in the jejunum and is evidence of the importance of the amino acid for broiler health. Methionine residues have been shown to be potent endogenous antioxidants that regulate the activity of biological proteins [15]. In addition to the direct antioxidant effect of methionine residues in proteins, the organism has a highly sophisticated enzyme system that minimizes the deleterious effects of oxidation. SOD and CAT are examples of important antioxidant enzymes.

In our study, the lowest expression levels of *SOD1* and *CAT* were observed in EC chickens. EC broilers fed the NS diet had lower CAT activity but higher SOD activity than EC broilers fed the *dl*-Met or *dl*-MMet diets. The reduction in *CAT* expression and consequently in CAT activity might have been caused by NO production and as a result of cellular damage in the jejunum [71, 72], which allows us to infer that the formation of toxic substances during the coccidial infection exceeded the antioxidant capacity of the defense system. This condition might have been further aggravated by methionine deficiency and by the fact that NO can inhibit the enzyme glutathione peroxidase [73]. On the other hand, higher SOD activity seems to be useful to combat the excess free radicals produced during the infection, resulting in a lower expression of the *SOD1* gene. Another possible explanation for the high SOD activity observed is the existence of cellular compensatory responses to the deficiency of methionine and other non-enzymatic antioxidants observed during the infection [11, 74]. Therefore, methionine deficiency and the inactivation of CAT can at least partially explain the lipid and protein oxidation observed in the jejunum of EC broilers fed the NS diet. Another interesting result of this study is that chickens fed the *dl*-Met and *dl*-MMet diets had lower nitrite content, which may be associated with the ability of methionine to react with RNS, thereby preventing cellular oxidation. This result highlights the importance of methionine, both as free amino acid and dipeptide, for the maintenance of intestinal epithelium. Furthermore, we observed that EC chickens fed the *dl*-MMet diet exhibited higher TAC, as did UC chickens fed the *dl*-Met and *dl*-MMet diets. These unprecedented results indicate that methionine dipeptide supplementation might play a key role in protecting the intestinal environment against oxidative stress in broilers challenged with *Eimeria* spp.

## Conclusions

We concluded that the poor performance observed in chickens challenged with *Eimeria* spp*.* probably occurred as a consequence of inflammatory responses and oxidative stress in the jejunum, which in turn might have affected intestinal epithelial integrity. The main effect of methionine supplementation was on antioxidant metabolism. Our results showed for the first time that free methionine and methionine dipeptide supplementation is able to protect the intestinal cells of broilers under *Eimeria* challenge from the oxidative damage induced by free radicals.

## Abbreviations

*ACTB*: Beta actin gene
ANOVA: Analysis of variance
*B*^*0*^*AT1*: Neutral amino acid transporter 1 gene
BWG: Body weight gain
*CAT–1*: Cationic amino acid transporter 1 gene
CAT: Catalase
*CAT*: Catalase gene
cDNA: Complementary DNA
*CLDN1*: Claudin-1 gene
CP: Carbonylated protein
*dl*-Met: free *dl*-methionine
*dl*-MMet: *dl*-methionyl-*dl*-methionine
DNA: Deoxyribonucleic acid
DNPH: 2,4-dinitrophenylhydrazine
DPPH: 2,2-diphenyl-1-picrylhydrazyl
EC: *Eimeria*-challenged
EDTA: Ethylenediaminetetraacetic acid
FCR: Feed conversion ratio
FI: Feed intake
H_2_O_2_: Hydrogen peroxide
HCl: Hydrochloric acid
*IFNG*: Interferon gamma gene
IL-10: Interleukin 10
*IL1B*: interleukin 1 beta gene
*IL2*: Interleukin 2 gene
*iNOS*: Inducible nitric oxide synthase gene
*LITAF*: Lipopolysaccharide-induced tumor necrosis factor (TNF)-alpha factor gene
MHA: Methionine hydroxy analog
MyD88: Myeloid differentiation primary response 88
NaNO_2_: Sodium nitrite
NCBI: National Center for Biotechnology Information
NED: N-(1-naphthyl) ethylenediamine dihydrochloride
ng: Nanogram
nm: Nanometer
nmol: Nanomo
NO: Nitric oxide
NO_2¯_: Nitrite
NO_3_^−^: Nitrate
NOS: Nitric oxide synthases
NS: Non-supplemented
O_2_: Superoxide anion
*OCLN*: Occludin gene
PAMP: Pathogen-associated molecular patterns
*PEPT1*: Peptide transporter 1 gene
PI: Post-inoculation
RNA: Ribonucleic Acid
RNS: Reactive nitrogen species
ROS: Reactive oxygen species
RT-qPCR: Reverse transcription polymerase chain reaction quantitative real time
SOD: Superoxide dismutase
*SOD1*: Superoxide dismutase 1 gene
spp.: Species
TAC: Total antioxidant capacity
TBARS: Thiobarbituric acid reactive substances
TJ: Tight junctions
TLR: Toll-like receptor
*TLR2*: Toll-like receptor 2 gene
*TLR5*: Toll-like receptor 5 gene
TNF: Tumor necrosis factor
TRIF: TIR-domain-containing adapter-inducing interferon-β
U: Unit
UC: unchallenged
*y*^*+*^*LAT-1*: Neutral and cationic amino acid transporter 1 gene
